# Prevalence and sensitization of atopic allergy and coeliac disease in the Northern Sweden Population Health Study

**DOI:** 10.3402/ijch.v72i0.21403

**Published:** 2013-08-05

**Authors:** Stefan Enroth, Ingrid Dahlbom, Tony Hansson, Åsa Johansson, Ulf Gyllensten

**Affiliations:** 1 Department of Immunology, Genetics and Pathology, SciLifeLab Uppsala, Rudbeck Laboratory, Uppsala University, Uppsala, Sweden; 2 Department of Womens and Childrens Health, Uppsala University Hospital, Uppsala University, Uppsala, Sweden; 3 Uppsala Clinical Research Center, Uppsala University Hospital, Uppsala University, Uppsala, Sweden

**Keywords:** allergy, coeliac disease, atopic allergy, heritability, self reported allergy

## Abstract

**Background:**

Atopic allergy is effected by a number of environmental exposures, such as dry air and time spent outdoors, but there are few estimates of the prevalence in populations from sub-arctic areas.

**Objective:**

To determine the prevalence and severity of symptoms of food, inhalation and skin-related allergens and coeliac disease (CD) in the sub-arctic region of Sweden. To study the correlation between self-reported allergy and allergy test results. To estimate the heritability of these estimates.

**Study design:**

The study was conducted in Karesuando and Soppero in Northern Sweden as part of the Northern Sweden Population Health Study (n=1,068). We used a questionnaire for self-reported allergy and CD status and measured inhalation-related allergens using Phadiatop, food-related allergens using the F×5 assay and IgA and IgG antibodies against tissue transglutaminase (anti-tTG) to indicate prevalence of CD.

**Results:**

The prevalence of self-reported allergy was very high, with 42.3% reporting mild to severe allergy. Inhalation-related allergy was reported in 26.7%, food-related allergy in 24.9% and skin-related allergy in 2.4% of the participants. Of inhalation-related allergy, 11.0% reported reactions against fur and 14.6% against pollen/grass. Among food-related reactions, 14.9% reported milk (protein and lactose) as the cause. The IgE measurements showed that 18.4% had elevated values for inhalation allergens and 11.7% for food allergens. Self-reported allergies and symptoms were positively correlated (p<0.01) with age- and sex-corrected inhalation allergens. Allergy prevalence was inversely correlated with age and number of hours spent outdoors. High levels of IgA and IgG anti-tTG antibodies, CD-related allergens, were found in 1.4 and 0.6% of participants, respectively. All allergens were found to be significantly (p<3 e–10) heritable, with estimated heritabilities ranging from 0.34 (F×5) to 0.65 (IgA).

**Conclusions:**

Self-reported allergy correlated well with the antibody measurements. The prevalence of allergy was highest in the young and those working inside. Heritability of atopy and sensitization was high. The prevalence of CD-related autoantibodies was high and did not coincide with the self-reported allergy.

Atopic allergy and coeliac disease (CD) are multi-factorial diseases caused by genetic and environmental factors. There is geographic variation in the occurrence of atopic diseases with an increasing prevalence in industrialized countries (1, 2), in particular among more recent birth cohorts (3). In addition, within countries, a higher proportion of individuals living in urban areas suffer from atopy as compared to those living in the rural parts (4, 5). The causative risk factors for the development of atopy have not been identified, but air pollution (6), climate change and socio-economic conditions, as well as the exposure to infectious agents in early childhood, have been suggested to influence the development (3, 7). It is generally accepted that allergic atopy mainly is Th2 mediated, with elevated levels of interleukin-4 (IL4), IL-5 and IL-13, in combination with the production of IgE antibodies against the afflicting allergens (8). The presence of IgE against specific allergens despite the lack of symptoms indicates sensitization, which is associated with a higher risk for future allergy development (9).

CD is caused by dietary gluten in genetically predisposed individuals carrying the human leukocyte antigen (HLA) DQ2 or DQ8 (10). Consumption of wheat gliadin induces an inflammation of the small intestine, with a destruction of the small intestinal mucosa as a result. The symptoms are frequently vague and span from a completely silent disease to overt enteropathy (11). A global prevalence around 1% has been reported (11), with an increased prevalence and incidence during the past decades (12). The incidence and prevalence differ slightly between and within countries (13, 14). The causes for the increasing prevalence of CD have not been clarified, but altered dietary habits and socio-economic conditions (15) have been suggested as environmental causative factors. CD is considered as an autoimmune, Th1-driven disease associated with elevated levels of interferon-γ, IL-2, IL-6 and tumour necrosis factor-α (16) and the production of IgA or IgG antibodies against tissue transglutaminase (tTG), an enzyme present in the small intestinal mucosa (17). Detection of anti-tTG antibodies is frequently used in the diagnostic routine to identify subjects with CD (18, 19). It has been suggested that Th1- and Th2-driven diseases are inversely related (20, 21), whereas conflicting results have been reported regarding the co-morbidity of allergy and CD (21–24).

Most studies on the prevalence of allergy and specific allergens, as well as the prevalence of CD, have been performed in rural areas, and there is a lack of information from more remote populations and, in particular, those living in arctic and sub-arctic areas. The northern parts of Norway, Sweden, Finland and the Kola Peninsula, collectively called the Northern Shield, are affected by both climate and cultural changes. These changes are causing a transition from a traditional subsistence-based lifestyle towards a modern industrialized lifestyle. The traditional lifestyle is based on reindeer herding, fishing and hunting, but other occupations are becoming more prevalent, causing a shift in diet, physical activity level and the amount of time spent outdoors (25). The full effect of this transition on disease prevalence is at present not known and requires special medical attention (26), and there is a need to focus on the health conditions of rural populations on the northern periphery of major continental areas. When rural populations in Nordic regions undergo lifestyle changes, many common diseases increase in prevalence (27). Studies of rural populations may therefore contribute to our understanding of common diseases. Rural populations have also been suggested to be important for studying complex traits, such as allergy (28).

The Northern Sweden Population Health Study (NSPHS) was conducted to provide a health survey of the communities of Karesuando and Soppero, County of Norrbotten, Sweden. The study was performed in 2 periods, with 750 participants mainly from the Karesuando area in 2006, and 350 participants from the Soppero and Vittangi area in 2009. The aim of the present study was to determine the prevalence of allergies in these communities and gain a better understanding of the environmental factors affecting allergic sensitization. To this end, we investigated the frequency of atopy-related IgE antibodies, specific allergens against food-related and inhalation-related atopy and the CD-related IgA and IgG anti-tTG antibodies. In addition, we examined the heritability of these estimates.

## Materials and methods

### Internal review board

The NSPHS study was approved by the local ethics committee at the University of Uppsala (Regionala Etikprövningsnämnden, Uppsala, Dnr 2005:325) in compliance with the Declaration of Helsinki (29). All participants gave their written informed consent to the study including the examination of environmental and genetic causes of disease. In case the participant was not full age, a legal guardian also signed. The procedure, which was used to obtain informed consent and the respective informed consent form, has been recently discussed in the light of present ethical guidelines (30).

### Allergens

Quantification of antibodies was made with an automated Phadia^®^ 250 system in accordance with the manufacturer's instruction. For detection of IgA and IgG anti-tTG antibodies, ELIA™Celikey IgA or ELIA™ Celikey IgG wells coated with recombinant human tTG were used. For determination of total IgE and allergen-specific IgE, ImmunoCAP^®^ anti-IgE, F×5 and Phadiatop were used. A full description of the methods used here is available in the Supplementary Material.

### Clinical symptoms of allergy

Participants were asked to fill out a questionnaire regarding their health condition and that of close relatives. This questionnaire had a section relating to allergy and allergic symptoms where the participant was asked to indicate the strength of symptoms on a scale from none, to strong (in 4 categories) for each of the following types of allergy: grass/pollen, nettle fever, eczema/rashes, breading/asthma, cow milk, gluten, fur, fish, dust, cold air, mould, organic solvents, medicines and other (specified). Any medicine used to treat an allergy was noted. The list of symptoms included running nose, coughing, swelling, hissing sound when breathing, difficulty breathing and dry skin, and the severity of symptoms was graded on a scale from none to strong (in 4 classes). Finally, the symptom for a food allergy was noted.

### Occupation

Participants of the study were asked in the questionnaire to specify their current employment. In this region, it is common to have several jobs depending on seasons, for example. This is especially evident among the reindeer herders. Consequently, the job-type stratification was done manually in strict groups of individual jobs predominately conducted indoors, outdoors or a mixture of both. In the material, there is also a large group working in the mine industry that is a very unique environment.

### Statistic analysis

The results from antibody measurements were analyzed using the R-language (31) using functionality from the GenABEL-package (32). Heritability was estimated using the polygenic function in the GenABEL-package based on the kinship between individuals. The kinship matrix were calculated using SNP data for 176,967 autosomal markers that were genotyped in all participants using the Illumina Infinium HumanHap300v2 or Illumina Omni Express SNP bead microarrays as described previously (33). A full description of the methods used here is available in the Supplementary Material.

## Results

### High prevalence of allergy

**Fig. 1:**
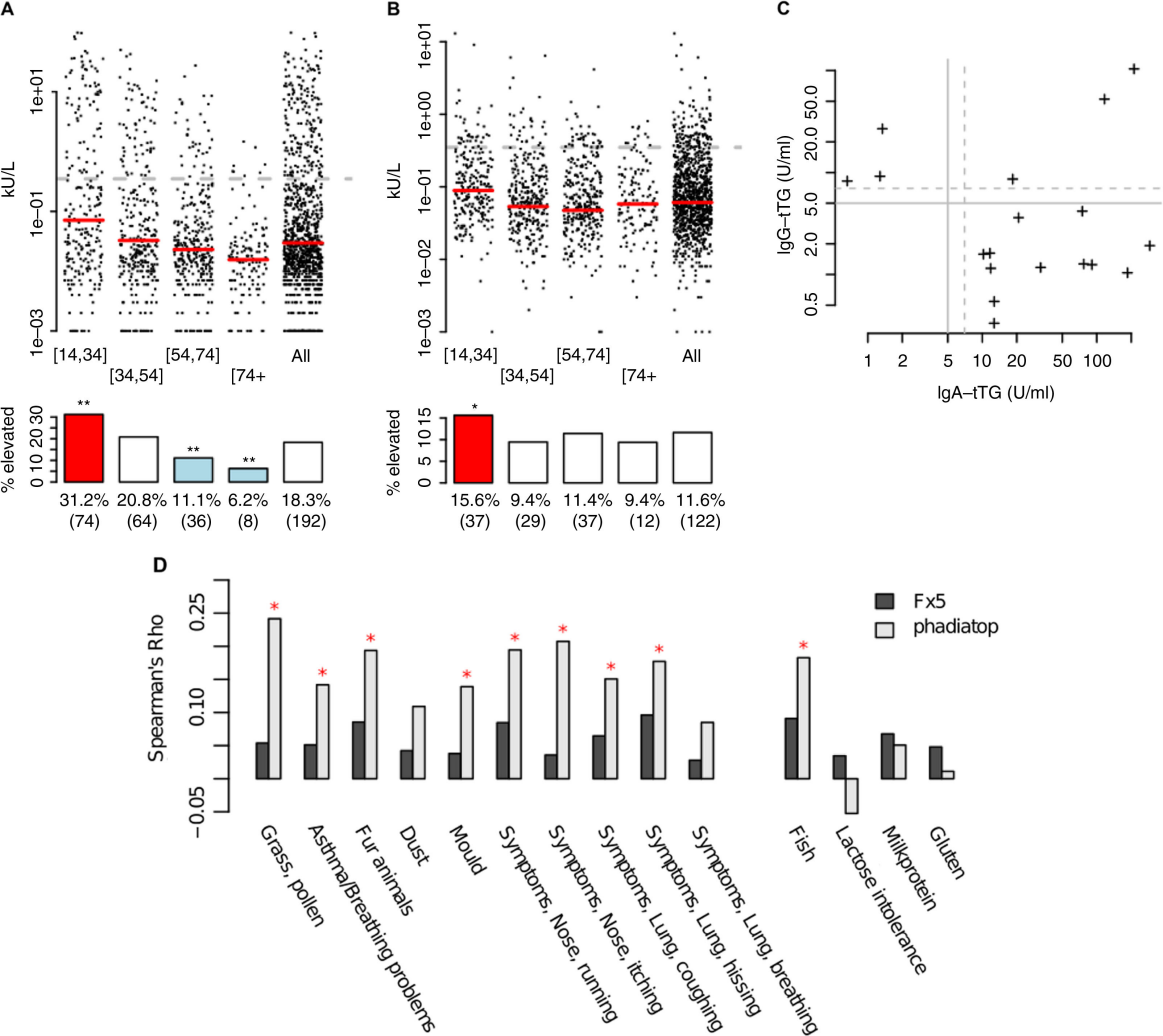
Antibody measurement stratified according to age correlations with self-reported data. (A) Phadiatop, (B) F×5 and (C) IgG-tTG vs IgA-tTG. Dotted lines indicate clinical cut-off for elevated values. For A and B, these represent 0.35kU/L. The ages are (left to right) 14–34 (n=237), 35–54 (n=307), 55–74 (n=324), 74+ (n=128) and all ages (n=1,048). A red (blue) coloured box depicts significantly (Fisher's exact test, *p<0.05, **p<0.001) enriched (depleted) number of elevated individuals compared to the whole cohort. (D) The IgE measurements have been adjusted for age, sex and BMI. Red coloured *indicated significant correlations (p<0.01).

The frequency of self-reported allergy symptoms was very high, with 42.3% (452/1,068) reporting mild to severe allergy symptoms (Table I). Among participants, 26.7% reported inhalation-related allergy, 24.9% food-related and 2.4% skin-related allergy. Of the inhalation-related allergies, 11.0% of the participants had reactions against fur and 14.6% against pollen/grass. Among food-related reactions, milk (protein and lactose) was the most frequent cause and reported by 14.9% of participants, followed by fish (3.3%) and gluten (1.9%). The *in vitro* tests (ImmunoCap allergy assays) showed lower frequencies of allergy, with 18.3% of the participants having elevated IgE against inhalation-related allergen antigens using the Phadiatop assay, and 11.6% having elevated levels of IgE against food-related allergens using the F×5 assay (Fig. 1A and B, respectively). Regarding CD-related antibodies, elevated IgA anti-tTG was found in 1.4% (15/1,068) and elevated IgG anti-tTG was found in 0.6% (6/1,068) of the participants (Fig. S1A and B, respectively). Three individuals had elevated levels of both IgA and IgG anti-tTG (Fig. 1C). Combined, 2.2% of the participants had either elevated IgA/IgG anti-tTG or self-reported gluten intolerance. Finally, increased total IgE was found in 6.4% of individuals (Fig. S1C).

**Table I:** Number of individuals with self-reported allergies

Allergy type			No. individuals	Percent of all	Percent in job-type category			

					In	Out	Mine	Herding
Any allergy			452	42.3	50.3	29.8	35.6	34.5
	Skin		26	2.4	3.7	1.6	0.0	1.4
	Inhalation		285	26.7	33.6	15.7	23.3	19.7
		Fur	117	11.0	13.9	3.7	2.2	4.2
		Dust	89	8.3	10.8	2.6	5.6	3.5
		Mould	100	9.4	11.9	2.6	8.9	4.9
		Pollen/Grass	156	14.6	18.0	9.4	13.3	13.4
	Food		266	24.9	30.6	15.7	16.7	19.7
		Fish	35	3.3	4.6	2.1	1.1	2.8
		Gluten	20	1.9	1.7	2.1	2.2	2.8
		Milk	159	14.9	16.5	11.0	14.4	14.8

All positive indications were used; only categories available from both questionnaires are reported.

### High correspondence between self-reported allergy and allergy tests

IgE levels measured with Phadiatop (adjusted for age and sex) were positively correlated with self-reported allergic symptoms elicited by grass/pollen, fur and mould. A significant correlation was observed between Phadiatop and numerous self-reported symptoms, such as asthma/breathing problems, running or itching nose, coughing and hissing (Fig. 1D). Interestingly, there was also a significant correlation between the self-reported allergies to fish and elevated IgE measured with Phadiatop, although this allergy panel does not include food-allergens. Self-reported food allergies showed a positive correlation with age- and sex-corrected reaction to food allergens as measured by the F×5 assay, although these did not reach statistical significance (p>0.01). When the material was stratified based on self-reported allergies, the measured levels of IgE, F×5 and Phadiatop allergens all showed higher median values in those with self-reported allergy as compared to the median for the entire material (Table SI). This was especially pronounced for self-reported inhalation-related allergies (Table SI), with the highest enrichment over population median for self-reported fur allergy and Phadiatop. Total IgE levels were enriched in all categories with inhalation-related allergens. As expected, almost all individuals with self-reported allergies against gluten had lower IgA and IgG anti-tTG levels as compared to the population median.

### Allergy is more common in young ages

We stratified the analysis on age by comparing 4 age groups (14–34, 34–54, 54–74 and 74+ years). Both Phadiatop and F×5 showed a significantly higher number of individuals with elevated levels in the youngest age group, as compared to the population median (Fig. 1A, B). For the inhalation-related allergens (Phadiatop), individuals with elevated IgE levels were enriched 2.5 fold in the 14–34 years group, as compared to the population median, and for the food-related allergens (F×5), elevated levels were enriched 1.5 fold in this age group (Table SII). There was also a significant over-representation of individuals in the youngest age group with elevated measurements, and also an overall shifted distribution of raw values toward higher levels (Table SII). The opposite pattern was found at the other end of the age spectrum, with individuals in the 74+ age group being under-represented among those with elevated allergen levels and lower raw values. This difference was not statistically significant. Both IgA and IgG anti-tTG levels were higher in the older age group than the younger group (Fig. S1A and B). In the analysis of medians, individuals with elevated IgA and IgG anti-tTG levels were enriched 1.1–1.2 fold among those aged 54–74 years, over the population median (Table SII).

### Trend towards lower inhalation-related allergy for those with outdoor occupations

Based on self-reported information on occupation, participants were classified as working predominantly indoors, outdoors or having a mixed working situation. A total of 78.8% of the participants fit into these categories. The bulk of the remainder was retired or students without steady work (labelled “unknown” below). We focused on 2 occupations specific to this region: mining and reindeer herding. There is a trend towards higher levels of atopic allergy for indoor jobs and lower levels for outdoor jobs, although no statistically significant differences were seen between different occupations after adjustment for age, sex and body mass index (BMI) (Table SIII). A significantly lower frequency of individuals with elevated IgE levels as measured with Phadiatop was observed in the groups with outdoors or mining occupations compared to the indoors group. The “retired” and “unknown” groups showed an opposite pattern for the Phadiatop measurements, which is explained by the age distribution in these groups

## Discussion

We have studied both self-reported allergy symptoms and measured IgE levels in a large population-based cohort from the very northern part of Sweden (Lat 68°3). The self-reported prevalence of allergy (42.3%) in this population is very high. Our allergy assays showed a prevalence of sensitization to inhalation-related allergens of 18.3% and to food-related allergens of 11.6%. Thus, whether we base the comparison on our data from self-reported allergy or on the allergy tests, the prevalence estimates for the northern Swedish population are unusually high. By comparison, a recent (2012) study reported a prevalence of self-reported allergy symptoms in the Stockholm area of 28.0% (men 26.6%, women 29.1%), and 33.6% in those aged 30–40 years, similar to estimates in a previous study conducted 10 years ago from the same region (34). Few studies are available from circumpolar populations. A study in Greenland based on pooled tests for specific IgE against the 8 most common inhalant allergens (grass, birch, mugwort, dog, cat, horse, *Cladosporum herbarum* and house dust mite) showed that the frequency of atopy has almost doubled from 10% in 1987 to 19% in 1998 (35). The increase was most pronounced in the youngest age group (15–19 years) but was also seen in older age groups, indicating that the risk factors responsible for the increase in atopy do not operate solely in childhood. A separate study on the prevalence of atopy in Inuit children living on the West coast of Greenland showed that 14.6% of the children were sensitized to at least one inhalant allergen and 4.1% to at least 1 food allergen (36). In this study, sensitization to grass was the most common, while the reaction to birch, animal-dander and house-dust mite was much more infrequent. In families where the parents were born abroad, the children had a higher frequency of sensitization to inhalant allergens as compared to children born to Greenlandic parents. These studies indicate that Greenlandic children have a lower prevalence of allergic sensitization towards inhalant allergens relative to the estimates from European studies. Our results suggest that the population of the northernmost part of Sweden have higher levels of allergic sensitization. This variation between populations may be due to difference in the genetic susceptibility to atopy, different exposure to allergens, or variation in the environment and living conditions.

In our study, the correlation between the self-reported allergy and sensitization measured by ImmunoCAP was high. A stronger correlation was seen between the inhalation-related sensitization results and the self-reported allergy and its symptoms, relative to the comparison for food-related allergens. This is to be expected, given the diffuse symptoms resulting from allergic reactions to food, which may be difficult to identify for the participants. A previous meta-analysis of food-allergens emphasized the large variability seen in the self-reported prevalence between studies, making it difficult to interpret differences observed between populations that are based solely on self-reporting (37). The meta-study also showed that the self-reported prevalence of food-related allergens is higher than the prevalence estimated based on objective assessments such as the skin prick test sensitization, sensitization assessed by serum IgE or by food challenge (37). This is consistent with the results of the present study. We found the food and respiratory atopy were significantly over-represented among individuals in the youngest age group. This is consistent with previous studies where individuals aged 20–39 have the highest prevalence of IgE-mediated food allergies (38). However, the prevalence in the oldest age groups is lower than in other (39). Since sensitization occurs during early childhood, our observation may reflect the living conditions of children growing up 50–70 years ago. We can only speculate about the causal factor, since this could include differences in a number of the exposures to specific allergens or to more time outdoors. To study the effect of spending time indoors and outdoors on the prevalence of inhalation-related allergy, we compared occupational groups and noted that there is an under-representation of strong allergic reactions among those having outdoor occupations. This is suggesting that the time spent outdoors is a strong contributing factor in allergy. This is most likely explained by differences in lifestyles, where younger persons tend to spend less time outdoors than older, especially on working days where our data has significant correlations (p<2 e–6) between age and time spent outdoors. We also noted a higher prevalence of self-reported allergy and sensitization to some of food allergens in the higher age group. Previous reports have suggested that food allergies are under-diagnosed among the elderly (40). The mechanism that alters the response/sensibilization in elderly compared to young is not known. The higher prevalence in the elderly may reflect alterations in food supplies or changes in the immune system with age, triggering the development of allergic reactions.

Given the nature of the study population, with a large number of family members included, we computed the heritability of the ImmunoCAP-based Phadiatop and F×5 estimates. Our estimated heritabilites were high: for F×5 0.34 (p<2.5 e–10), Phadiatop 0.35 (p<1 e–16), IgG 0.38 (p<5 e–14), IgE 0.53 (p<1 e–16) and for IgA 0.65 (p<1 e–16). In a previous study, based on the concordance in monozygotic and dizygotic twins, the concordance rates for total IgE and specific IgE to Der p 1, mixed grass pollen and cat dander were also very high, corresponding to an estimated heritability of 0.60 (41). This confirms that genetic factors are important in susceptibility to inhalation-related allergen sensitization and allergic disease. Since many of the monozygotic twins were discordant for atopy, exposure to a number of environmental factors is also needed for the development of the symptoms

CD is accompanied by elevated serum levels of autoantibodies against tTG, and IgA anti-tTG and IgG anti-tTG antibodies were measured in all individuals in order to investigate the potential occurrence of CD. The prevalence of CD in the western world is at least 1%, and the true prevalence of CD is likely to be even higher (42). These frequencies vary over the European continent with the notably higher frequencies found in Finland (2.4%) as compared to Italy (0.7%) (13). In the present study, 2.2% of the participants had either elevated anti-tTG autoantibodies or self-reported gluten intolerance. One explanation for the high prevalence might be that the population studied has an unusually high genetic risk of CD. On the other hand, we could have made an overestimation of the prevalence due to the fact that not all our antibody-positive subjects have undergone the biopsy procedure recommended for confirmation of CD beyond doubt. Interestingly, almost all individuals with self-reported allergies had lower serum levels of anti-tTG antibodies as compared to the population median and we could not find any individuals with co-existing diseases.

In summary, we found a high degree of sensitization to common allergens in the studied population, and the highest allergy prevalence was found in the youngest individuals and among participants mainly working indoors. Also, the prevalence of CD was high and did not coincide with the self-reported allergy. The heritability of atopy was high, and further studies concerning allergy and genetic susceptibility in rural Nordic populations are needed.
